# High-Resolution Depth Profiling of Residual Stresses in PVD Coatings on Additively Manufactured Polymers via FIB-DIC and Eigenstrain Theory

**DOI:** 10.3390/ma19061171

**Published:** 2026-03-17

**Authors:** José Daniel Rodríguez-Mariscal, Karuna Srivastava, Ismael Romero-Ocaña, Ramón Escobar-Galindo, Andrea Bernasconi, Jesús Hernández-Saz

**Affiliations:** 1 Department Continuum Mechanics and Structural Analysis, University of Seville, Camino de los Descubrimientos s/n, 41092 Seville, Spain; 2 Department of Mechanical Engineering, Politecnico di Milano, Via La Masa 1, 20156 Milano, Italyandrea.bernasconi@polimi.it (A.B.); 3 Department of Engineering and Materials Science and Transportation, University of Seville, Camino de los Descubrimientos s/n, 41092 Seville, Spain; jhernandez32@us.es; 4 Department of Materials Science and Metallurgical Engineering and Inorganic Chemistry, University of Cadiz, Campus Universitario Río San Pedro s/n, Puerto Real, 11510 Cádiz, Spain; ismael.romero@uca.es; 5 Department of Applied Physics I, University of Seville, Virgen de África 7, 41011 Sevilla, Spain; rescobar1@us.es

**Keywords:** residual stresses, PVD coatings, additive manufacturing, polymers, FIB-DIC, eigenstrain theory, methodological optimization, polymer-metal interface

## Abstract

The synergy between additively manufactured (AM) polymers and functional PVD coatings is crucial for advanced applications, yet the reliability of these hybrid systems is dictated by the residual stresses induced during deposition. This work presents the first in-depth, nanoscale profiling of residual stresses in Ti6Al4V and SS316 coatings on 3D-printed Acrylonitrile Styrene Acrylate (ASA) and Silicon (Si) substrates. A cutting-edge methodology combining Focused Ion Beam (FIB) milling with Digital Image Correlation (DIC), rigorously interpreted through the non-integral eigenstrain theory, is employed. Our findings reveal a consistent pattern of compressive stresses near the coating surface but expose a significant tensile stress peak at the coating-substrate interface, a feature not observed on reference silicon substrates. High-resolution electron microscopy and elemental analysis suggest that this stress concentration is associated with the presence of a thin, brittle oxide interlayer formed on the substrate surface. Furthermore, this study quantifies the dominant effect of the low-stiffness polymer substrate, which leads to a strain relief magnitude an order of magnitude higher than in rigid substrates. This work provides critical quantitative data on the failure-driving mechanisms in these emerging material systems and establishes a robust, optimized metrological protocol for their characterization.

## 1. Introduction

Additive Manufacturing (AM) has catalyzed a transformation in materials engineering, enabling the production of polymeric components with optimized geometries and a complexity unattainable by subtractive methods [1]. This capability has opened the door to innovations in fields as diverse as bioengineering, with personalized implants, or the aerospace industry, with lightweight components [2]. Specifically, Fused Filament Fabrication (FFF) allows for cost-effective manufacturing of thermoplastic materials such as Acrylonitrile Styrene Acrylate (ASA), known for its weather resistance and mechanical stability [3,4]. However, for many of these high-performance applications, the intrinsic properties of the polymers are insufficient. This is where functional coatings, applied via techniques such as Physical Vapor Deposition (PVD), play a crucial role, endowing the surfaces with properties like wear resistance, biocompatibility, electrical conductivity or electromagnetic shielding (EMI) [5,6,7,8]. PVD is often the preferred method due to its compatibility with the low thermal budget required by polymer substrates [9].

Despite their advantages, the combination of a rigid, dense coating on a soft, lightweight polymeric substrate is a formidable engineering challenge. During the PVD process, a series of physical mechanisms—ranging from high-energy ion bombardment to rapid post-deposition cooling—induce a complex state of residual stresses (RS) in the thin film [10,11,12]. These stresses, if tensile, can facilitate crack propagation, while if excessively compressive, they can cause buckling and delamination of the coating. Considering that delamination is a critical failure mode in soft substrate systems, the magnitude and profile of these stresses through the coating thickness are, therefore, determinant factors for adhesion, mechanical integrity, and ultimately, the service life of the component.

The characterization of these RS in hybrid polymer systems is notoriously difficult. Non-destructive techniques such as X-ray Diffraction (XRD), standard for crystalline materials, are limited by the amorphous or semi-crystalline nature of polymeric substrates and the small interaction volume of nanometric coatings. This limitation is further compounded by the impossibility of using flexure-based methods or traditional hole-drilling techniques due to the low stiffness and poor spatial resolution they offer. On the other hand, macroscopic destructive methods (such as drilling or cutting) lack the spatial resolution necessary to resolve stress gradients across a thickness of only a few hundred nanometers.

To overcome these limitations, the micro-machining technique using a Focused Ion Beam (FIB) combined with Digital Image Correlation (DIC) has become established as the method of choice for RS evaluation at the micro and nanoscale [13,14]. The “ring-core” methodology, in particular, allows for the isolation of a material micropillar, whose surface relaxation, measured with nanometric precision by DIC, contains the information of the pre-existing stress state. However, meticulous control is required to mitigate potential artifacts like ion beam damage.

The final step, the conversion of the measured strain data into a stress profile, has also been subject to significant evolution. The classical approach, based on an integral deconvolution [15], is an ill-conditioned inverse problem, prone to numerical instabilities. A paradigm shift was the introduction of the eigenstrain (autodeformation) theory by Korsunsky et al. [16]. This theoretical framework establishes a non-integral and local relationship between the measured strain and the stress source (the eigenstrain), drastically simplifying the reconstruction problem and increasing its robustness [17,18]. This non-integral approach is paramount for accurately reconstructing steep stress gradients, particularly those expected at the coating-substrate interface.

Nevertheless, the application of this sophisticated methodology to soft polymeric substrate systems is not without challenges. The significant elastic contrast between the stiff coating and the compliant substrate, combined with the potential surface roughness inherited from the AM process and the thermal sensitivity of the polymer to the ion beam, demand meticulous control and optimization of every stage of the experimental and data analysis procedure.

This work presents an integral study applying the FIB-DIC/eigenstrain methodology to Ti6Al4V and SS316 coatings on ASA and Ti substrates, compared against reference Silicon substrates. It addresses methodological challenges by establishing a rigorous protocol [19], quantifies the soft substrate effect, and suggests the brittle oxide interlayer as a likely dominant failure driver.

Nevertheless, the application of this sophisticated methodology to soft polymeric substrate systems is not without challenges. The significant elastic contrast between the stiff coating and the compliant substrate, combined with the unique surface roughness and topological constraints inherited from the AM process, demand meticulous control and optimization of every stage of the experimental and data analysis procedure.

While FIB-DIC has been applied to standard smooth polymers, its application to AM substrates remains underexplored. This work bridges this gap by presenting an integral study applying the FIB-DIC/eigenstrain methodology to Ti6Al4V and SS316 coatings on ASA and Ti substrates, compared against reference Silicon substrates. It addresses methodological challenges by establishing a rigorous protocol [19], quantifies the soft substrate effect, and suggests the brittle oxide interlayer as a likely dominant failure driver.

## 2. Materials and Methods

### 2.1. Sample Fabrication

Two types of substrates were used: (i) Acrylonitrile Styrene Acrylate (ASA) fabricated using FFF with a layer height of 0.2 mm and 100% infill; and (ii) monocrystalline Silicon wafers as a rigid reference material. The FFF samples were printed with a 0/90° build orientation to minimize anisotropy effects on the plane of measurement.

Two types of coatings were deposited onto these substrates using PVD via the “magnetron sputtering” technique. The deposition parameters for both coatings are summarized in Table 1. The coatings used were:

- Ti6Al4V: Biomedical grade titanium alloy [20].
- Stainless Steel 316 (SS316): Austenitic steel with high corrosion resistance [21].

The coating thicknesses, subsequently measured by TEM, ranged from 540 to 1275 nm. The PVD process was carried out in a specific system.

### 2.2. Microstructural and Chemical Characterization

The coating microstructures and interfacial integrity were analyzed using Transmission Electron Microscopy (TEM) and Scanning Transmission Electron Microscopy (STEM) in an FEI Talos F200X system (Thermo Fisher Scientific, Waltham, MA, USA) operated at 200 kV. Site-specific electron-transparent lamellae for TEM analysis were prepared and extracted in situ using a Thermo Fisher Scientific Scios 2 DualBeam FIB-SEM system (Thermo Fisher Scientific, Waltham, MA, USA). Furthermore, the elemental composition distribution across the interface was mapped using Energy Dispersive X-ray Spectroscopy (EDX) (Thermo Fisher Scientific, Waltham, MA, USA) coupled with the TEM.

### 2.3. Theoretical Framework and Numerical Calibration

#### 2.3.1. The Eigenstrain Approach vs. Integral Methods

The relationship between the observed surface strain relief, e(h), and the residual stress profile, σ(z), has traditionally been described by a first-kind Volterra integral equation [15], while mathematically sound, solving this equation constitutes an ill-posed inverse problem. Small uncertainties in the experimental strain data are amplified during the integral inversion, frequently resulting in non-physical oscillations in the calculated stress profile, particularly at greater depths.

To overcome these limitations, the eigenstrain theory was adopted [16]. This approach postulates that the total residual elastic strain (ε) is the additive sum of the strain relief (*e*), related to stress via Hooke’s law, and the inelastic eigenstrain ($\epsilon^{*}$), which represents the source of the residual stresses.

(1) $$\varepsilon =e+\epsilon^{*}$$

For the specific geometry of the FIB ring-core milling, Korsunsky et al. demonstrated that the relationship between the measured surface strain and the underlying eigenstrain at a specific depth *h* can be decoupled into a local, non-integral form [16]:

(2) $$\epsilon^{*}\left(h\right)=\frac{de(h/D)/d(h/D)}{F(h/D)}$$

Here, *D* is the central pillar diameter, *h* is the milling depth, and F(h/D) is the “Master Influence Function.” This function acts as a depth-dependent calibration factor, quantifying the sensitivity of the surface strain to the removal of a stressed layer at depth *h*. Unlike the integral method, this local derivative approach significantly reduces noise amplification and allows for a more robust reconstruction of sharp stress gradients.

#### 2.3.2. Generalization for Non-Equibiaxial Stress States

Since PVD coatings may exhibit anisotropic stress states, the scalar formulation is generalized by decomposing the strain tensor into its hydrostatic (volumetric) and deviatoric (shear) components. For each milling increment *i*, the incremental strain relief (Δe) is separated into hydrostatic ($\Delta e_{H,i}$) and deviatoric ($\Delta e_{D,i}$) parts:

(3) $$\Delta e_{H,i}=\frac{\Delta e_{I,i}+\Delta e_{II,i}}{2},\;\Delta e_{D,i}=\frac{\Delta e_{I,i}-\Delta e_{II,i}}{2}$$

Two distinct influence functions, $F_{H}$ (hydrostatic) and $F_{D}$ (deviatoric), are required to relate these strain components to the source eigenstrains ($\epsilon^{*}$):

(4) $$\left[\begin{array}{c} \epsilon_{I,i}^{*}\\ \epsilon_{II,i}^{*} \end{array}\right]=-\frac{1}{F_{H}\left(h_{i}\right)}\left[\begin{array}{c} \Delta e_{H,i}\\ \Delta e_{H,i} \end{array}\right]-\frac{1}{F_{D}\left(h_{i}\right)}\left[\begin{array}{c} \Delta e_{D,i}\\ -\Delta e_{D,i} \end{array}\right]$$

Finally, the residual stress profile is calculated from the reconstructed residual elastic strains using Hooke’s law for plane stress conditions, as follows: $\varepsilon =-\epsilon^{*}$ [16,17].

#### 2.3.3. Finite Element Calibration of Influence Functions

The accuracy of the stress reconstruction depends entirely on the validity of $F_{H}$ and $F_{D}$. Given the significant elastic contrast between the coating and the ASA substrate, generic functions cannot be used. Therefore, specific functions were calibrated using 3D Finite Element Modeling (FEM) in ABAQUS/CAE 2024 (Dassault Systèmes, 2023).

**Figure 1:**
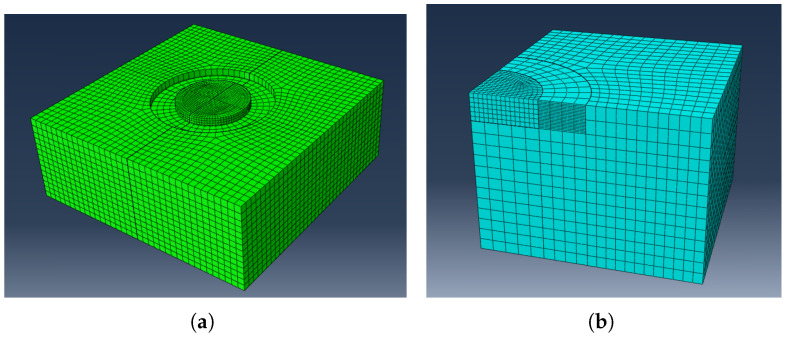
Finite Element Model used for calibration: (**a**) 3D geometry of the ring-core system and (**b**) Quarter-symmetry mesh details showing high element density in the pillar and trench region using C3D8 elements.

A quarter-symmetry model of the pillar-substrate system was constructed using C3D8 linear hexahedral elements (Figure 1). To ensure numerical accuracy, a mesh convergence study was performed. The element size in the critical pillar region was progressively refined from 500 nm down to 100 nm. It was observed that reducing the local seed size below 150 nm resulted in a variation in the calculated strain relief of less than 2%. Consequently, a local seed size of 150 nm was selected as the optimal balance between accuracy and computational cost.

The elastic properties of the coatings and substrates used for the calibration of the influence functions are summarized in Table 2. The values for ASA, Si, SS316, and Ti6Al4V were obtained from the Granta Edupack database, while the properties for TiN were taken from literature [22]. All materials were assumed to exhibit linear elastic behavior during the milling simulation.

To determine $F_{H}$ and $F_{D}$, unit eigenstrain fields were simulated: (1) Hydrostatic Mode: A uniform equi-biaxial strain field ($\epsilon_{x}=\epsilon_{y}=1\%$) applied to the boundaries; and (2) Deviatoric Mode: A pure shear strain field ($\epsilon_{x}=-\epsilon_{y}=1\%$). The material removal process was simulated by sequentially deactivating elements in the annular trench region (element birth/death technique). At each step, the average relaxed strain was extracted from the nodes on the pillar surface, restricted to the central 80% of the radius to match the experimental ROI.

The resulting cumulative strain relief curves, f(h/D), were fitted using an empirical exponential decay function:

(5) $$f\left(h/D\right)=1-\exp\left(-a\frac{h}{D}\right)\left[1+b\left(\frac{h}{D}\right)-c{\left(\frac{h}{D}\right)}^{2}+d{\left(\frac{h}{D}\right)}^{3}\right]$$

**Figure 2:**
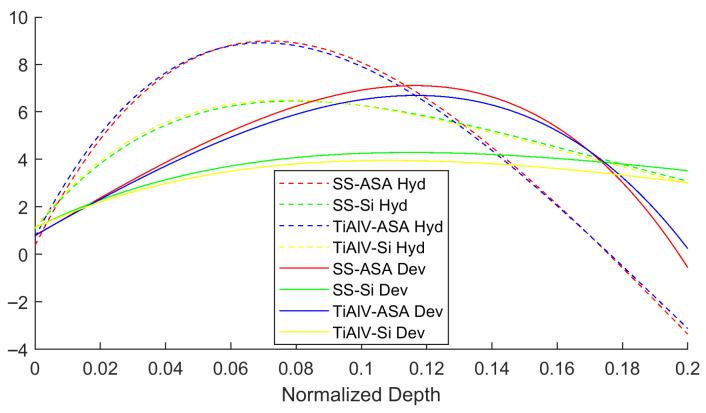
Calibrated Master Influence Functions ($F_{H}$ and $F_{D}$) derived from FEM. The distinct curves for ASA and Silicon substrates highlight the necessity of substrate-specific calibration due to the stiffness mismatch.

The Master Influence Functions were then obtained by analytically differentiating these fitted curves (F=df/d(h/D)), as shown in Figure 2. This rigorous calibration ensures that the specific mechanical interaction between the rigid coating and the compliant ASA substrate is correctly accounted for in the stress calculation.

### 2.4. Residual Stress Measurement via FIB-DIC

#### 2.4.1. Milling and Image Acquisition Protocol

**Figure 3:**
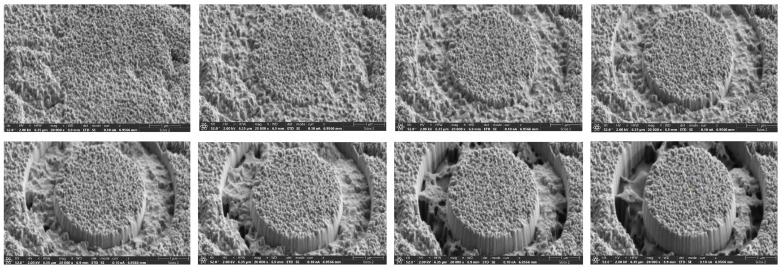
Representative FIB-DIC milling sequence showing the progressive material removal for residual stress analysis.

Measurements were performed using an Thermo Fisher Scientific Scios 2 DualBeam FIB-SEM system. The ring-core geometry was employed [13]. An annular trench was milled incrementally, isolating a central micropillar of 3 μm diameter. The pillar diameter (*D*) was selected for each sample such that the ratio between the maximum milling depth ($h_{max}$, equal to the coating thickness) and the diameter was maintained in the optimal range of $h_{max}/D<0.2$ to maximize measurement sensitivity [17]. A low gallium ion current (10–30pA) was used to minimize surface damage and ion implantation ensuring minimal impact on non-targeted zones. Figure 3 shows SEM images during the annular material removal process, which leaves the island in the center that will be analyzed by DIC.

After each milling increment (typically 20–30 nm), the process was stopped, and a high-resolution SEM image of the pillar surface was acquired. This process was repeated until the trench depth exceeded the total coating thickness, ensuring a complete relaxation of the stresses contained within it.

#### 2.4.2. Optimization of Digital Image Correlation (DIC) Analysis

**Figure 4:**
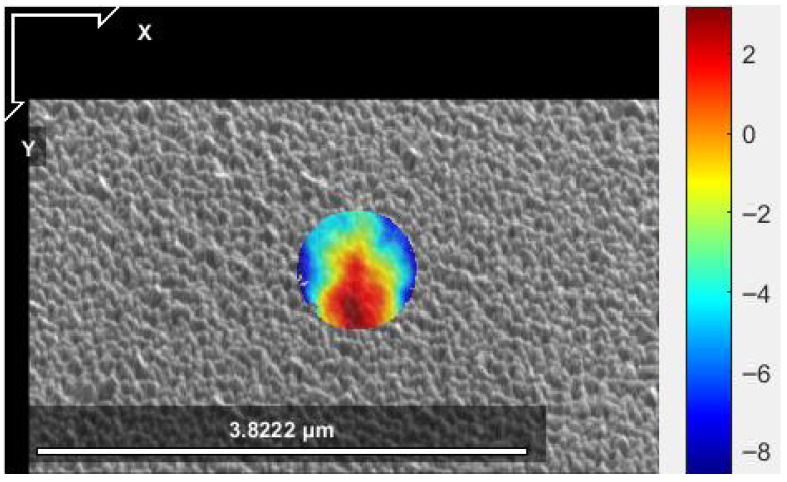
Example of optimization of DIC parameters: (**Left**) Correlation coefficient distribution [-]; (**Right**) Green-Lagrangian shear strain field ($\varepsilon_{xy}$) [-].

The reliability of the FIB-DIC technique is fundamentally dependent on the quality and stability of the SEM image analysis. Consequently, a systematic methodological study was performed to optimize the DIC parameters using the open-source software NCORR in MATLAB [23]. This optimization, described in detail in [5], followed the best practice guidelines for FIB-DIC established by the National Physical Laboratory (NPL) [19]. A critical outcome of this study concerned image resolution; it was determined that a resolution set to 70% of the maximum capacity provided the optimal balance between spatial accuracy and algorithm stability, as illustrated in Figure 4. Higher resolutions tended to prevent robust convergence of the DIC algorithm due to excessive data noise, while lower resolutions compromised the necessary spatial precision for nanoscale measurements.

To ensure the integrity of the strain data, the selection of the Region of Interest (ROI) was strictly controlled. Since the FIB milling process inevitably induces peripheral damage and ion-beam-related artifacts at the edge of the trench, the ROI was restricted to the central 80% of the micropillar area. This exclusion of the damaged outer zone proved crucial for stabilizing the measurements and significantly reducing the dispersion of the strain results [19]. Furthermore, an intensity averaging filter was applied to the entire image sequence prior to analysis. This pre-processing step effectively minimized electronic noise and improved the signal-to-noise ratio, which was found to be particularly beneficial for the stainless steel samples.

Finally, specific DIC engine parameters were standardized to guarantee consistent tracking throughout the milling increments. A subset radius of 70 pixels combined with a step of 5 pixels was employed to provide sufficient overlap for reliable correlation. To handle the complex relief patterns observed in these hybrid systems, the convergence algorithm was allowed a maximum of 100 iterations. This optimized configuration ensured robust displacement tracking even across the steep strain gradients typically encountered at the coating-substrate interface.

## 3. Results

### 3.1. Microstructure of Coatings and the Interface

**Figure 5:**
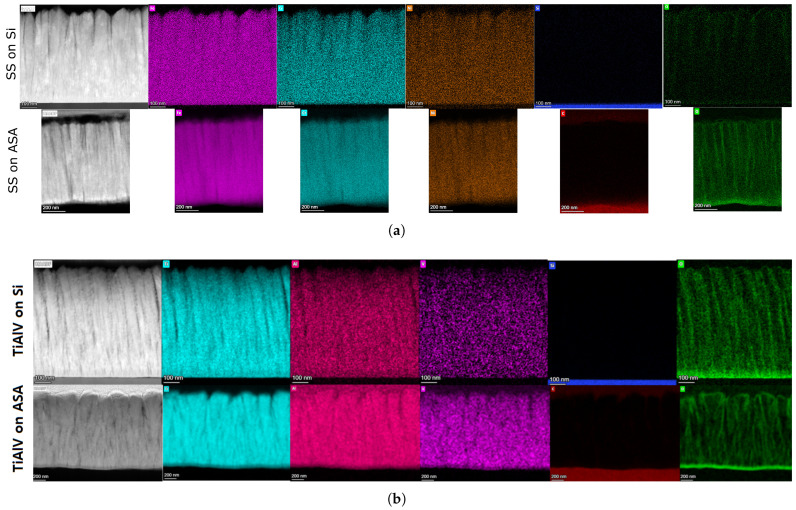
Cross-sectional electron microscopy images of (**a**) SS316 and (**b**) TiAlV coatings deposited on Silicon (top row) and ASA (bottom row) substrates, highlighting the microstructural differences. Note the columnar growth structure is maintained on both substrates, but the interface roughness is higher on the polymeric substrate.

TEM analysis revealed that both Ti6Al4V and SS316 coatings exhibited a columnar microstructure characteristic of PVD processes, with column widths ranging between 50 and 105 nm (Figure 5). High-resolution TEM (HRTEM) confirmed that the coatings possess a mixed structure, with nano/microcrystalline domains embedded within an amorphous matrix. Cross-sectional STEM-EDX elemental mapping (Figure 5) demonstrated a homogeneous distribution of the principal metallic constituents throughout the entire coating thickness for all samples. Ti, Al, and V were uniformly distributed in the Ti6Al4V coatings, while Fe, Cr, and Ni showed consistent composition across the SS316 films on both substrate types. The most distinctive microstructural feature observed was the presence of an oxygen-rich interlayer at the coating-substrate interface. EDX oxygen mapping (Figure 5, green channel) revealed a thin layer, 5–10 nm thick, located precisely at the interface in all ASA-based samples (both Ti6Al4V/ASA and SS316/ASA) and in Ti6Al4V/Si sample. The coating microstructures remained columnar and continuous across the interface in all cases, with no evidence of delamination, voiding, or gross interfacial disruption.

**Figure 6:**
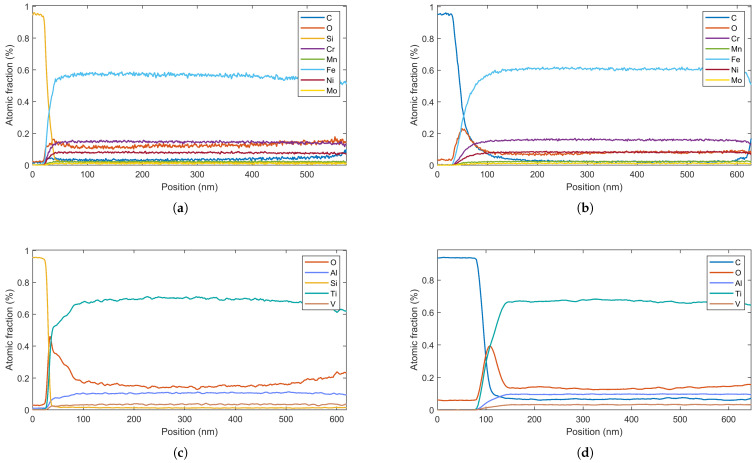
EDX elemental concentration profiles across the coating-substrate interface for: (**a**) SS316/Si; (**b**) SS316/ASA; (**c**) Ti6Al4V/Si; (**d**) Ti6Al4V/ASA. The red line highlights the specific accumulation of oxygen at the interface of the polymeric samples, absent or negligible in the silicon references.

Figure 6 presents EDX elemental concentration profiles measured perpendicular to the coating surface. For all samples, the metallic constituents exhibited nearly constant atomic percentages throughout the coating thickness. However, pronounced oxygen concentration peaks (red curves) were systematically detected at the interface in three sample types: SS316/ASA (Figure 6b), Ti6Al4V/Si (Figure 6c), and Ti6Al4V/ASA (Figure 6d), reaching local concentrations exceeding 20 at.%.

### 3.2. Strain Relief Curves: The Substrate Stiffness Effect

**Figure 7:**
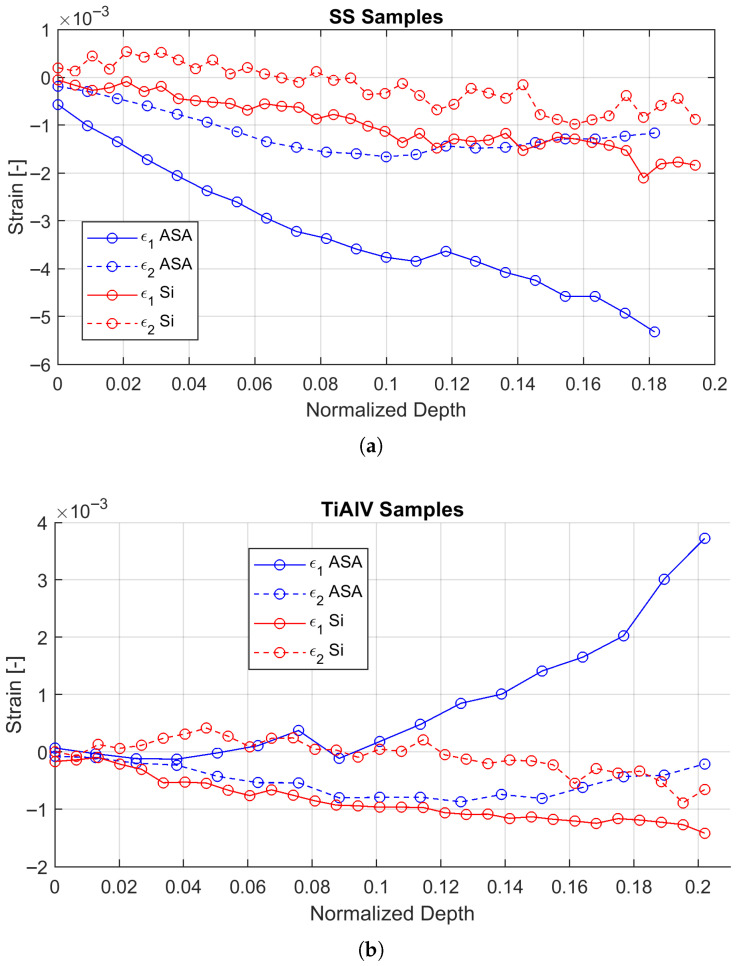
Comparison of the principal surface strain relief curves ($\epsilon_{1}$ and $\epsilon_{2}$) for the coatings on ASA (blue lines) and Silicon (red lines) substrates as a function of normalized milling depth: (**a**) Ti6Al4V samples; (**b**) SS316 samples. The plots show a significantly greater strain magnitude for the polymeric substrate. The difference highlights the critical need for substrate-specific Influence Functions calibrated via FEM.

Figure 7 compares the strain relief curves for the SS316 coating on an ASA substrate and on Silicon. The magnitude of the total strain released was an order of magnitude greater in the ASA substrate (polymer) compared to the rigid Silicon substrate. This result quantitatively confirms the effect of the low stiffness of the polymer, which allows for a much more pronounced relaxation of the coating upon removal of the constraint by FIB milling. This phenomenon, while expected, is quantified here for the first time and has a significant practical implication: the FIB-DIC technique is inherently more sensitive and requires less deep milling steps to capture full relaxation on soft substrates. Figure 7 illustrates the surface strain relief evolution obtained from DIC. For the SS316 samples, the strain relief is predominantly negative, indicating that the coating was in a tensile residual stress state prior to milling (relief is compressive). As expected, the magnitude of the strain relief on the compliant ASA substrate is significantly higher than that on the rigid Si substrate.

Conversely, for the Ti6Al4V samples, the strain relief is positive, indicating a compressive residual stress state. Consistent with the SS316 results, the magnitude of relaxation is much larger on the polymer substrate. This order-of-magnitude difference highlights the critical influence of substrate stiffness ($E_{sub}$) on the mechanical equilibrium of the system.

### 3.3. Residual Stress Profiles

**Figure 8:**
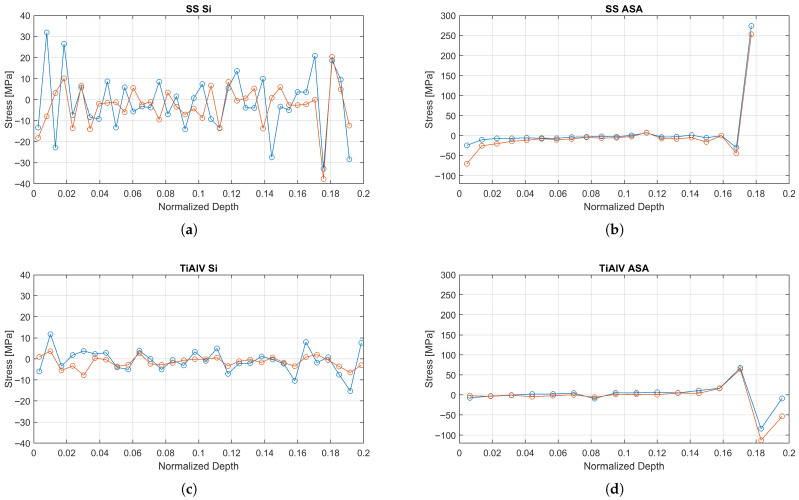
Depth-resolved principal residual stress profiles reconstructed via the eigenstrain method. The profiles show $\sigma_{1}$ (blue circles) and $\sigma_{2}$ (orange circles) for (**a**) Ti6Al4V/ASA, (**b**) SS316/ASA, (**c**) Ti6Al4V/Si, and (**d**) SS316/Si. The ASA samples exhibit a pronounced stress concentration at the coating-substrate interface (normalized depth ≈ 0.18–0.2), whereas the coatings on Silicon show a more stable, lower-magnitude stress distribution.

The reconstructed stress profiles are presented in Figure 8. All coatings on ASA substrates showed a compressive stress state near the surface (attributed to atomic peening [12]), followed by a sharp gradient towards a tensile stress peak at the interface.

The reconstructed residual stress profiles reveal a consistent and well-defined mechanical behavior for all coatings deposited on ASA substrates. In all cases, the two principal stress components ($\sigma_{1}$ and $\sigma_{2}$) were nearly identical throughout the coating thickness, confirming that the stress state is predominantly equibiaxial. Near the coating surface, all systems exhibited a compressive residual stress state, with values ranging from approximately −25 MPa for the TiAlV coating to about −70 MPa for SS316. Moving towards the coating–substrate interface, a sharp stress gradient was systematically observed, leading to the development of a pronounced tensile stress peak precisely at the interface (normalized depth = 1). The magnitude of this interfacial tensile peak was strongly dependent on the coating material, reaching approximately +275 MPa for SS316 on ASA and around +70 MPa for TiAlV on ASA. This interfacial tensile stress concentration arises from the combined effect of thermo-elastic mismatch between the coating and the polymer substrate and the presence of the brittle oxide layer identified in Section 3.1, which acts as an efficient stress concentrator and is therefore considered the most likely driver for failure initiation in both SS316 and TiAlV coatings.

## 4. Discussion

The combination of microstructural and mechanical results allows for the construction of a coherent image of the behavior of these hybrid systems.

The systematic presence of an oxygen-rich interlayer at specific coating-substrate interfaces represents a critical finding for understanding the mechanical behavior and failure mechanisms of these hybrid systems. Notably, this oxide layer was observed in all ASA-based samples (SS316/ASA and Ti6Al4V/ASA) and in Ti6Al4V/Si, but was absent in SS316/Si. This finding is consistent with previous studies on metallized polymers, where distinct substrate oxide layers have been identified at the interface [24].

For both Ti6Al4V/ASA and SS316/ASA, the oxide interlayer formation is primarily attributed to plasma-induced surface oxidation of the ASA substrate during the early stages of PVD. Prior to metal atom arrival, the substrate surface is exposed to the high-energy plasma environment inherent to magnetron sputtering, which contains reactive oxygen species from the residual atmosphere and target surface oxides. It is well-established that plasma exposure of polymer surfaces promotes several simultaneous phenomena: (i) chain scission and formation of radical sites; (ii) incorporation of oxygen-containing functional groups (C=O, C-O-C, -OH); and (iii) formation of a modified surface layer with altered stoichiometry [24,25]. McClure et al. demonstrated that evaporated aluminum on polypropylene develops interfacial oxide layers with thicknesses directly proportional to oxygen plasma treatment levels, ranging from 2 to 15 nm [24]. Similarly, Yun et al. identified that oxide coatings on plasma-damaged polymers form a chemically distinct interlayer that significantly affects both coating adhesion and microstructure [25].

The presence of an oxide interlayer in Ti6Al4V/Si but not in SS316/Si reveals a coating-material-specific mechanism. Titanium and its alloys have exceptionally high oxygen affinity and readily form stable oxides (${\mathrm{TiO}}_{2}$, ${\mathrm{Al}}_{2}O_{3}$) even at low oxygen partial pressures [26]. During the initial stages of Ti6Al4V deposition, the arriving metal atoms can react with residual oxygen in the chamber and with the native ${\mathrm{SiO}}_{2}$ on the silicon surface, promoting the formation of a mixed Ti-Al oxide interlayer. The thickness of this layer (around 40 nm) significantly exceeds that of native ${\mathrm{SiO}}_{2}$ (∼2 nm), indicating active oxidation during early-stage growth.

The oxide layer introduces an additional interface with distinct elastic properties. For ASA substrates, the metal-polymer interface already exhibits a stiffness ratio of ∼50–100; the insertion of a semi-rigid oxide creates two sequential interfaces with sharp property gradients. For Si substrates with Ti6Al4V, the oxide creates a stiffness gradient between the coating and substrate, with the oxide acting as an intermediate phase. This configuration is known to promote stress concentration and is less tolerant of interfacial shear than a smooth bimaterial transition [27].

### 4.1. Combined Influence of Substrate Stiffness and the Interface

The marked difference in strain relief between the ASA and Silicon substrates (Figure 7) is a direct manifestation of their stiffness mismatch. The ASA substrate, with a Young’s modulus of ≈2 GPa [1], acts as a “soft” foundation that offers little constraint to the coating (whose modulus is ≈110–200 GPa). This stiffness ratio ($E_{film}/E_{sub}\approx 100$) implies that the boundary conditions for stress accumulation are fundamentally different from rigid substrates, allowing for significant substrate compliance even under residual loads. This has two consequences: it modifies the stress state stored within the coating and, simultaneously, facilitates the measurement of its relaxation by providing a larger strain signal.

The most significant finding is the spatial correlation between the tensile stress peak observed in the RS profiles (Figure 8) and the oxygen-rich layer detected by EDX at the interface, while direct mechanical testing of this 5–10 nm layer is beyond the scope of this work, elastic mismatch theory [27] suggests that the insertion of a semi-rigid oxide layer between a compliant polymer and a stiff metal creates a zone of stress concentration. This brittle interlayer likely acts as a “weak link,” unable to accommodate the shear strains generated by the thermal mismatch, thereby serving as a potential initiation site for delamination.

### 4.2. Interpretation of Stress Profiles

The typical stress profile observed in the samples on ASA (compression at the surface, tension at the interface) is the result of a competition between intrinsic and thermal stress-generating mechanisms. Ion bombardment during PVD induces strong compressive stress in the surface layers (atomic peening) [11].

Regarding the interfacial tensile peak, while thermal mismatch typically induces compression upon cooling (as the polymer shrinks more than the metal), the observed tensile stress is likely driven by the island coalescence mechanism (Volmer-Weber growth) during the initial stages of deposition [12]. During nucleation, the metallic islands exert tensile forces to close the gaps between them. As the film thickens, this mechanism is overtaken by the compressive atomic peening, resulting in the observed steep gradient through the thickness. This gradient is further exacerbated by the presence of the oxide interlayer, which may prevent effective stress relaxation at the polymer surface.

### 4.3. Critical Importance of Methodological Optimization

This study underscores that obtaining reliable results in complex material systems depends fundamentally on methodological rigor. The sensitivity analysis performed on the DIC parameters [5] is not merely a technical appendix, but a necessary condition for the validity of the conclusions. Without the exclusion of the outer 20% of the ROI, the interfacial stress peak could have been masked by milling artifacts [19]. Similarly, without the selection of an optimal image resolution, the acquisition of displacement data itself could have failed due to lack of convergence. Thus, the protocol established here serves as a validated guideline for future FIB-DIC investigations on soft or additively manufactured substrates [14,19].

### 4.4. Sensitivity Analysis and Uncertainty Quantification

Given the semi-destructive nature of the FIB-DIC technique, statistical validation via repetition on the same sample is not possible. To address the robustness of the calculated stress profiles, a sensitivity analysis was performed regarding two critical factors: the definition of the Region of Interest (ROI) and the assumed elastic properties.

#### 4.4.1. Sensitivity to Input Parameters

The eigenstrain reconstruction relies on the quality of the strain data extracted from the ROI. We analyzed the impact of varying the ROI radius. Including the full pillar radius (100% ROI) introduces significant noise due to ion-beam-induced damage at the trench edges. However, restricting the ROI to the central 80% stabilizes the strain data. The variability of the calculated stress profiles within this stable regime was found to be approximately ±20 MPa. This uncertainty band is significantly lower than the magnitude of the tensile peaks observed (e.g., +275 MPa), confirming that the interfacial stress concentration is a statistically significant feature and not an artifact of data processing.

#### 4.4.2. Influence of Coating Stiffness

A limitation of this study is the use of bulk elastic properties for the PVD coatings. It is well-documented that nanometric PVD films may exhibit a Young’s modulus 10–30% lower than the bulk material due to porosity and columnar grain boundaries [12]. Since the eigenstrain method relies on linear elasticity ($\sigma =E\cdot \varepsilon_{elastic}$), a reduction in the coating modulus $E_{film}$ results in a linear scaling of the residual stress magnitude. If we assume a “worst-case” scenario where the PVD coating modulus is 30% lower than the bulk value used (e.g., ≈80 GPa for Ti6Al4V instead of 114.5 GPa), the calculated peak tensile stress at the interface would decrease proportionally (e.g., from +70 MPa to ≈+49 MPa). However, crucially, the sign and the gradient of the stress profile remain unchanged. Therefore, while the absolute quantitative values represent an upper bound based on bulk properties, the identification of the dangerous tensile state at the interface remains valid regardless of the specific modulus value.

## 5. Conclusions

This study successfully applied the FIB-DIC eigenstrain methodology to characterize the depth-resolved residual stress profiles of Ti6Al4V and SS316 coatings on additively manufactured ASA substrates. The main conclusions are:

1. Substrate Compliance Effect: The low stiffness of the AM polymer substrate governs the mechanical response, resulting in strain relief magnitudes an order of magnitude higher than those observed on rigid silicon reference substrates. This necessitates substrate-specific finite element calibration for accurate stress reconstruction.
2. Interfacial Stress State: Unlike the compressive state typically desired in PVD coatings, the systems on ASA exhibited a sharp stress gradient transitioning to a significant tensile peak at the coating-substrate interface (+275 MPa for SS316 and +70 MPa for Ti6Al4V).
3. Role of Interfacial Oxide: The tensile stress peak spatially coincides with a brittle interfacial oxide layer formed during the initial stages of deposition. This layer introduces a tertiary mechanical interface that likely acts as a stress concentrator, compromising adhesion.
4. Methodological Robustness: Sensitivity analysis confirmed that while variations in coating elastic properties scale the stress magnitude, the tensile nature of the interfacial stress is a robust feature of these hybrid systems.

These findings indicate that to improve the reliability of coated AM polymers, deposition strategies must focus on minimizing substrate surface oxidation and managing the thermal history to reduce interfacial tensile stresses.

To further advance this field, several research avenues are suggested. First, performing direct measurements of the thin film elastic properties via nanoindentation would reduce uncertainty in the stress values. This is critical, as the Young’s modulus of nanometric PVD films often deviates significantly from bulk literature values, directly scaling the calculated stress magnitude.

Additionally, exploring alternative validation techniques, such as cross-sectional synchrotron X-ray nano-diffraction, could provide independent verification of the stress profiles, provided the grain size allows for sufficient diffraction volume. Developing more sophisticated FEM models that explicitly include the interfacial oxide layer and the anisotropy of the columnar microstructure would also allow for a more realistic simulation. Finally, conducting a parametric study to investigate the influence of PVD parameters (e.g., bias voltage, substrate temperature) and 3D printing parameters (e.g., layer orientation, infill density) on the evolution of residual stresses would be valuable for process optimization.

## Figures and Tables

**Table 1:** PVD process parameters for TiAlV and SS316 coatings.

Sample	Power	Ar Flow	Pressure	Time	Thickness
TiAlV	100 kW	25 sccm	7.5 × $10^{-3}$ mbar	60 min	606 nm
SS316	100 kW	25 sccm	7.5 × $10^{-3}$ mbar	60 min	582 nm

**Table 2:** Mechanical properties of the materials used in the Finite Element Modeling (FEM) simulations.

Material	Young’s Modulus (*E*) [GPa]	Poisson’s Ratio (ν)
ASA	1.93	0.41
Silicon (Si)	159.0	0.27
SS316	197.0	0.27
Ti6Al4V	114.5	0.34

## Data Availability

The original contributions presented in this study are included in the article. Further inquiries can be directed to the corresponding author.
